# A multistage framework for respiratory disease detection and assessing severity in chest X-ray images

**DOI:** 10.1038/s41598-024-60861-6

**Published:** 2024-05-29

**Authors:** Pranab Sahoo, Saksham Kumar Sharma, Sriparna Saha, Deepak Jain, Samrat Mondal

**Affiliations:** 1 Department of Computer Science & Engineering, Indian Institute of Technology Patna, Patna, 801106 India; 2 Maharaja Surajmal Institute of Technology, Delhi, India; 3 Mount Sinai Hospital and Icahn School of Medicine, New York, USA

**Keywords:** Diseases, Health care, Medical research

## Abstract

Chest Radiography is a non-invasive imaging modality for diagnosing and managing chronic lung disorders, encompassing conditions such as pneumonia, tuberculosis, and COVID-19. While it is crucial for disease localization and severity assessment, existing computer-aided diagnosis (CAD) systems primarily focus on classification tasks, often overlooking these aspects. Additionally, prevalent approaches rely on class activation or saliency maps, providing only a rough localization. This research endeavors to address these limitations by proposing a comprehensive multi-stage framework. Initially, the framework identifies relevant lung areas by filtering out extraneous regions. Subsequently, an advanced fuzzy-based ensemble approach is employed to categorize images into specific classes. In the final stage, the framework identifies infected areas and quantifies the extent of infection in COVID-19 cases, assigning severity scores ranging from 0 to 3 based on the infection’s severity. Specifically, COVID-19 images are classified into distinct severity levels, such as mild, moderate, severe, and critical, determined by the modified RALE scoring system. The study utilizes publicly available datasets, surpassing previous state-of-the-art works. Incorporating lung segmentation into the proposed ensemble-based classification approach enhances the overall classification process. This solution can be a valuable alternative for clinicians and radiologists, serving as a secondary reader for chest X-rays, reducing reporting turnaround times, aiding clinical decision-making, and alleviating the workload on hospital staff.

## Introduction

Respiratory diseases, including tuberculosis (TB)^1^, pneumonia^2^, and COVID-19^3^, are widespread and pose substantial health risks. Timely detection and screening are crucial to halting disease progression and enhancing the effectiveness of treatment. Chest X-rays (CXRs) play a pivotal role in the initial assessment of these conditions, where expert radiologists analyze images to pinpoint regions of infection. However, the resemblance in CXR findings among COVID-19, pneumonia, and TB patients complicates the interpretation process, making it labor-intensive, time-consuming, and susceptible to errors. WHO reports a shortage of doctors, with fewer than one available for every 1000 people in over 45% of countries globally. The onset of the COVID-19 pandemic in December 2019 further exacerbated this situation, affecting millions and disrupting the patient-doctor ratio. This makes each medical professional examine a huge number of radiography images, increasing the likelihood of human error with subjective differences^1^. In recent years, the advancement of Deep Learning (DL) has been instrumental in various applications. Convolutional Neural Networks (CNNs) have shown remarkable success in speech recognition, object identification, image recognition, and machine translation tasks. CNNs excel at learning hierarchical visual features when provided with ample training data. However, acquiring extensive datasets in the medical domain remains a challenge. Researchers utilized the concept of transfer learning, where models are trained on a large-scale dataset like the ImageNet^4^, and then pre-trained weights are considered for a small target dataset. In order to support clinical decisions, many Computer-Aided Diagnosis (CAD) systems have been developed. These systems are becoming increasingly common in various radiology contexts, including workflow optimization, abnormality detection, and disease progression monitoring^1,5–7^. Additionally, CAD-based solutions can offer doctors additional insights into the issue that might not be visible to the human eye. These solutions can prove invaluable in underserved areas and communities with limited access to trained radiologists.

### Related work

Detecting the abnormality and finding the infected regions in CXR images is vital to adopt a suitable treatment plan. However, it is difficult to pinpoint the infection’s exact location since various abnormalities share the same kinds of patterns. To address this issue, the authors in^8^ introduced a new dataset containing 112,120 CXR images of 14 distinct abnormalities. They utilized a weakly supervised approach to generate a likelihood of infection map. However, the Grad-CAM generated infection map does align accurately with the actual bounding box indicated in the ground truth. A related study by Rajpurkar et al.^5^ used DenseNet-121 to develop novel architecture and reported a better performance than the prior baseline method presented in^8^. Several other works^9,10^ also reported better performance using different advanced CNN models. After the outbreak of the recent pandemic in 2019, research efforts have redirected toward computer-aided detection (CAD) of respiratory diseases, including COVID-19^11–14^. The authors in^3^ developed a new dataset for COVID-19 detection from pneumonia and normal CXR images and presented a novel CNN-based model. Nishio et al.^15^ incorporated one private and two public CXR datasets and utilized transfer learning using EfficientNet B5 for classifying COVID-19, non-COVID-19, and healthy cases using CXR images. These approaches primarily use whole CXR images for model training, collected from diverse institutions employing different imaging devices and settings. This variation introduces discrepancies in radiographic attributes such as lighting, contrast, and resolution, ultimately resulting in poor generalization. To address this, few studies^1,14,16,17^ have incorporated lung segmentation before classification. The methods mentioned above employed one or a few segmentation models, choosing the best-performing model for final predictions. However, this approach has a significant risk of information loss, as selecting any single ’best’ model may only partially capture the complexity and diversity of the data. Another drawback of these works is that they mainly used data from the Montgomery^18^ and Shenzhen^18^ datasets, encompassing a range of CXR images with varying quality from both normal and Tuberculosis (TB) patients. Consequently, the segmentation performance tends to be suboptimal when dealing with severe COVID-19 cases exhibiting bilateral consolidation or fluid accumulation. This limitation significantly affects the system’s ability to classify such cases accurately. Moreover, earlier works have shown that a specific model can provide better results under particular conditions, highlighting the difficulty in achieving generalization^13,14^. In response, previous research efforts^19–22^ have utilized basic ensemble methods such as majority voting and weighted averaging.

The authors in^23^ presented a framework utilizing three pre-trained models and utilized an ensemble learning for COVID-19 detection from CXR images. However, these basic ensemble methods do not utilize the confidence scores of the classifiers during the final prediction computation, resulting in lower performance. In prior research on COVID-19 classification, few advanced fuzzy ensemble approaches have been explored^24–27^. Kundu et al.^28^ proposed an ensemble technique using the Sugeno fuzzy integral (SFI) and Dey et al.^29^ utilized Choquet fuzzy ensemble and achieved an accuracy of 99.02% on COVID-19 dataset. Additionally, the authors in^12^ presented a rank-based ensemble technique by employing two non-linear functions and reported 98.05% accuracy on a three-class classification problem. Beyond disease classification, identifying the infection areas and assessing the severity level is crucial for understanding a patient’s condition and guiding suitable interventions. This information assists in monitoring disease advancement, optimizing the allocation of scarce medical resources, and strategizing clinical treatments effectively. However, studies addressing severity scoring in CXR images are limited^30–32^. For instance, Mercaldo et al.^33^ introduced a deep learning based technique for COVID-19 detection from CT-scan images and automatically highlights symptomatic areas in the CT images, enhancing diagnostic precision. Cohen et al.^34^ utilized a severity rating system introduced in a previous work by Warren et al.^32^, assigning scores from 0-4 based on infection severity. Another researcher^30^ developed an array-based approach for severity assessment. This method divided the lung regions into several subdivisions and assigned scores ranging from 0-3. Another author in^35^ created and validated a severity assessment model encompassing COVID-19, influenza, and novel influenza, utilizing CT images from a diverse multi-center dataset and reported that the developed model also applies to patients with other types of viral pneumonia. In the proposed architecture, we have employed the modified the RALE scoring system introduced^12,32^ to assess COVID-19 CXR image severity. A literature review summary is presented in the Supplementary File.

### Contributions

To address the challenges outlined previously, we present a multi-stage architecture for disease classification (Pneumonia, TB, Normal, and COVID-19), identification of infection areas, and evaluating severity levels in COVID-19 patients. This research significantly advances the current state-of-the-art automated CXR image analysis, providing crucial support to medical professionals in diagnosing respiratory-related conditions.

- We have proposed a multi-stage pipeline designed explicitly for lung segmentation, disease classification, infection region identification, and severity assessment from COVID-19 CXR images.
- We employed several segmentation models to extract pertinent lung regions. The outputs from the top three models are combined and fed into the subsequent classification models.
- Most studies have only used two or three disease categories: normal, pneumonia, and COVID-19. However, tuberculosis, which shares similarities with these respiratory diseases, can sometimes coexist with COVID-19. We have considered both experiments in the proposed work.
- This study chooses a robust approach by utilizing three distinct CNN models rather than depending on a single classification model followed by the Gamma function for the ensemble.
- In the third stage of our proposed network, we incorporated an infection segmentation module to pinpoint the specific regions affected by the infection.
- Finally, we employed a severity assessment method utilizing the modified RALE scoring system. This approach allows for a detailed classification of COVID-19 CXR samples into distinct severity levels, including mild, moderate, severe, and critical.

## Problem formalization

In our proposed method, we have developed a multi-stage architecture to tackle the challenges encountered in CAD-based systems. In the first phase, the segmentation models identify the pertinent lung areas. In the subsequent step, classification models categorize these identified lung sections into distinct classes, followed by a Gamma-based fuzzy ensemble (GFE). Considering the dataset $D = {{\{D^i\}}_{i=1}^N}$, *N* is the total samples. Segmentation masks corresponding to each image $M = {\{M^i\}}_{i=1}^N,$ where $M^i$ is the individual segmented image. The output from the three classification models is ensembled using the Gamma function and mathematically formulated as X = GFE $[\{C_1(M^i)\},\{C_2(M^i)\},\{C_3(M^i)\}]$. Here, $C_1$, $C_2$, and $C_3$ are three pre-trained classifiers, and X is the predicted class.

## Dataset and methods

### Dataset statistics

Normal and pneumonia CXR images are collected from the Kaggle repository ”Chest X-Ray Images (Pneumonia)”^36^. The COVID-19 images are accumulated from another Kaggle repository^37^. It is noteworthy that the images in this repository originated from diverse sources, including some low-quality images with questionable labels. To ensure data reliability, we curated 4966 COVID-19 CXR images. This curation involved the removal of low-resolution images where COVID-19 patterns were not clearly visible and excluding CXR images with multiple lung diseases (multi-label) by an experienced radiologist. 700 TB CXR images are collected from the Kaggle repository^1^, a collection of two publicly available datasets: the Montgomery and Shenzhen^18^. For lung segmentation, we have employed the COVID-QU-Ex dataset^17^, the most extensive publicly accessible dataset related to COVID-19, along with the Montgomery and Shenzhen datasets. Furthermore, infection localization is pivotal for evaluating a patient’s condition and determining appropriate treatment strategies^38^. We utilized 2951 COVID-19 infection segmentation data from the study^39^, which included annotated ground-truth infection segmentation masks. Table 1 specifies detailed resources of the datasets.

**Table 1:** Summary of dataset resources.

Datasets	Classes	Images
(a) Classification dataset		
Kermany et al.^36^	Normal	1583
Kermany et al.^36^	Pneumonia	4273
Zhao et al.^37^	COVID-19	4966
Rahman et al.^1^	TB	700
(b) Lung segmentation and infection segmentation dataset		
COVID-QU-Ex dataset^17^	Normal	10701
	COVID-19	11956
(Lung segmentation)	Non-COVID	11263
Montgomery^18^	Normal/TB	138
Shenzhen^18^	Normal/TB	566
QaTa-COV19^39^	COVID-19	2913
(Infection segmentation)	–	–

### Methods

To develop a robust end-to-end architecture, we hypothesized the following:

#### Hypothesis 1

The Gamma-based ensembles may serve as multi-expert recommendations and lower the likelihood of incorrect diagnoses.

#### Hypothesis 2

The performance of the classification model may be enhanced by segmenting the lung areas in the initial stage.

#### Hypothesis 3

The proposed architecture’s infection segmentation module could improve the model’s interpretability rather than the Grad-CAM-based approach.

**Figure 1:**
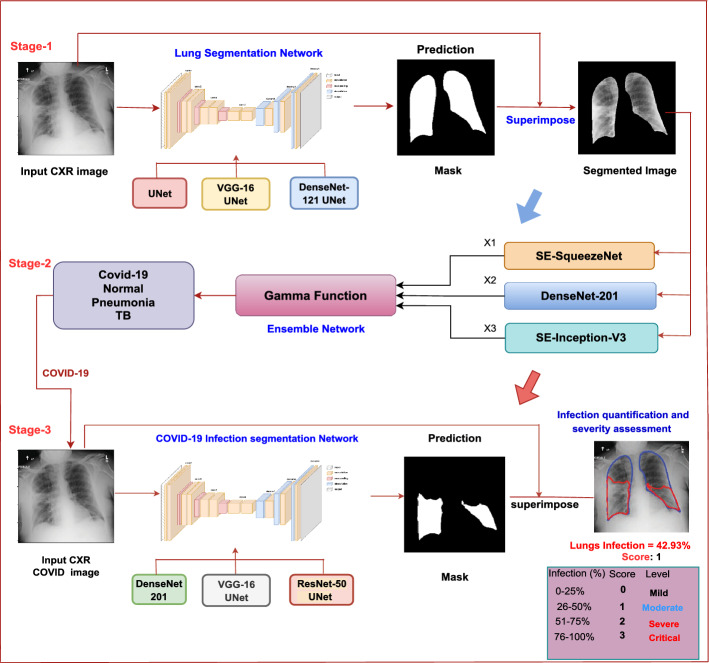
Block diagram of the proposed architecture. Stage 1 involves lung segmentation, Stage 2 focuses on disease classification, and Stage 3 incorporates infection segmentation and severity assessment.

We proposed a multi-stage architecture to evaluate the hypothetical assumptions, as shown in Fig. 1. The suggested approach comprises three main steps: lung segmentation, classification, and infection region segmentation with severity assessment. The following sections provide explanations for each level.

### Stage-I: segmentation

The proposed network’s first stage is extracting the relevant lung regions from the CXR images. CXR images acquired through different techniques often undergo varying compression methods, and different patients have different heart sizes, bone structures, cardio-thoracic ratios, and lung borders. These variations can introduce variability in the training process. The primary purpose of lung segmentation in the initial stage is to filter out irrelevant features, ensuring a more precise interpretation and generalization of the model in the subsequent classification phase. In our proposed network, we have utilized the UNet model^40^ along with six distinct backbone models: Attention, Inception-V3, DenseNet-201, ResNet-50, VGG-16, and DenseNet-121. After evaluating the performance (refer to Table 2), we have selected UNet-DenseNet121, UNet, and UNet-VGG16 for the final ensemble based on Intersection over Union (IoU) and Dice scores. All the input images are resized to 256$\times$256 as CXR images often come in varying sizes. Resizing the images to a specific resolution ensures uniformity and reduces computational complexity during processing. Next, we have normalized the image pixel values to a [0, 1] range, which enhances the model’s stability during training by preventing gradient explosion or vanishing during backpropagation. We also have utilized data augmentation techniques such as rotation, flipping, scaling, and translation to increase the diversity of the training dataset. Rotation involved randomly rotating the images within a angle range, flipping included horizontal and vertical flips, scaling encompassed random scaling of the images, and translation involved random shifts in the image position. Refer to the Supplementary File for the input CXR samples, the corresponding ground truth, the segmented masks, and the ensemble lung masks.

### Stage-II: classification models

The second stage classifies the segmented CXR images into specific classes based on the relevant lung regions. We have performed two classification experiments: one with a three-class (COVID-19, Normal, and Pneumonia) and another with a four-class (COVID-19, Normal, Pneumonia, and TB). We have adopted Inception-V3^41^ and SqueezeNet^42^ with Squeeze and Excitation (SE) block^43^ and DenseNet-201^44^ based on initial experiments. The reason for selecting SE-Inception-V3 is its depth and ability to capture complex image patterns. It uses a combination of convolutional layers with different kernel sizes, allowing it to capture features at various scales. SE-SqueezeNet is chosen for its lightweight architecture. It achieves good accuracy with significantly fewer parameters compared to traditional architectures. DenseNet-201 is chosen for its densely connected layers, facilitating feature reuse across layers. This architecture has shown to be very effective in learning intricate patterns from data, making it suitable for tasks where detailed feature extraction is crucial.

#### Proposed ensemble approach for final classification

Ensemble techniques are one of the most powerful techniques for decision-making that fuse several base classifiers’ confidence scores to arrive at the final results. It improves the entire model’s performance and removes the bias introduced by the base models. Recently, there has been an increase in the integration of ensemble learning with DL-based architecture^20,27,28^ like simple averaging, majority voting, and weighted average. However, these approaches have disadvantages like giving equal weight to each classifier irrespective of the performance, assigning a higher weight to classifiers with higher accuracy, and ignoring a group of classifiers working together^45^. In this study, we develop a rank-based ensemble approach utilizing the Gamma function to overcome these restrictions and solve the classification problem. This technique has the advantage of using adaptive weights, which prioritize the scores of the individual base classifiers while constructing the ensemble to produce the final confidence score (prediction) for each input sample.

Here, we provide an overview of the fuzzy Gamma-based ensemble techniques used to estimate confidence scores from the base learners. The Gamma function is a mathematical equation introduced by Leonard Euler^46^ and is defined as an extension of a factorial function for non-integral numbers. The Gamma function for a positive integer n and a complex number with a positive real part is defined as Eqs. (1) and (2), respectively.

1 $$\begin{aligned} \Gamma (n)&= (n-1)! \end{aligned}$$

2 $$\begin{aligned} \Gamma (\alpha )&= \int _{0}^{\infty } t^{\alpha -1}e^{-t} \,dt \end{aligned}$$

In this context, $\alpha$ is a parameter defined for all complex numbers except non-positive integers, and *t* is a variable representing the values over which the integral is performed. We calculate the rank of input images belonging to class k of the $i^{th}$ classifier’s confidence score (CS) as defined in Eq. 3.

3 $$\begin{aligned} R^i_k= \Gamma (CS^i_k), \forall i, k; i = 1,2,...,M; k = 1,2,...,K \end{aligned}$$

We have utilized three CNN models, so M = 3 and k = 3 or 4, depending on the number of classes. We calculate the fuzzy rank sum (FRS) and the complement of confidence factor sum (CCFS) for class k using Eqs. (4) and (5). We calculate the final decision score (FDS) for a class k using the below Eq. (6).

4 $$\begin{aligned} FRS_k= \sum _{i=1}^{M} R^i_k \end{aligned}$$

5 $$\begin{aligned} CCFS_k&= \frac{1}{M}\sum (1-CS_k)^i \end{aligned}$$

6 $$\begin{aligned} FDS_k&= FRS_k \times CCFS_k \end{aligned}$$

**Figure 2:**
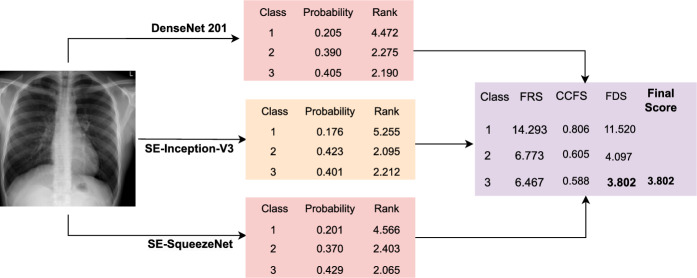
Mathematical example of the proposed ensemble approach. Initially, rank scores are computed from the three base CNNs. These scores are then fused together to generate an overall score, determining the final classification outcome.

The final predicted class for an image I is determined by Eq. 7, by computing the minimum FDS values. To enhance comprehension of the ensemble method, a hypothetical example is illustrated in Fig. 2.

7 $$\begin{aligned} class(I)=argmin_k(FDS_k) \end{aligned}$$

### Stage-III: infection segmentation and severity assessment

Identifying infection regions and assessing severity in real-time is paramount in clinical settings, offering vital insights into disease progression and guiding appropriate medical interventions. Upon categorizing the input image as COVID-19, it undergoes infection segmentation. Infection segmentation masks for COVID-19 images are only publicly available and utilized to train the COVID-19 infection segmentation models. We employed the UNet model^40^ with six different backbone networks similar to those used in lung segmentation. The infected regions are determined by combining the output of the top-performing models, namely UNet-DenseNet-201, UNet-ResNet50, and UNet-VGG16, selected based on IoU and Dice scores. The severity of lung infections is determined by calculating the percentage of infected pixels in the lungs. We have employed modified RALE scoring and devised a scale from 0 to 3 based on the extent of infection^12,32^. In the initial step, after lung segmentation, we identified the largest contour with OpenCV and overlaid it onto the original images. Subsequently, we segmented infection regions, detected contours using OpenCV, and superimposed them onto the lung mask contour. The infection percentage was then computed by dividing the pixels representing the overlap areas by the total lung mask pixels and multiplying by 100. For instance, if the infection involves less than 25% of the whole lung, it is classified as ”mild” (score 0). A score of 1 suggests involvement of less than 50% of the lung, indicating a ”moderate” severity. A score of 2 represents infection covering up to 75%, categorized as ”severe”. A score of 3 suggests extensive infection, from 76% to 100%, categorizing it as ”critical”. This scoring system provides a clinical grading for COVID-19 patients, enabling differentiation into distinct severity levels.

## Results and analysis

The outcomes of lung segmentation are presented in Table 2, and it is evident that no single model exhibits superior performance. To mitigate information loss during segmentation, we merged the mask outputs from the top three models (shown in bold). Quantitative analysis demonstrates a substantial enhancement in the ensemble lung mask, with a 3.13% increase in IoU and a 1.75% in the Dice score compared to the best-performing Dense121-UNet. The Adam optimizer with an initial learning rate $=0.0001$ is utilized, and an early stopping strategy, limiting the training of each model to a maximum of 100 epochs with a batch size of 8.

To show the effectiveness of the classification models, we have performed several experiments: 3-class and 4-class classification (with and without lung segmentation). Table 3 represents the outcome of 3-class classification models. SE-Inception-V3 demonstrated a superior performance among the three models in the initial experiment conducted without lung segmentation and attained an accuracy of 97.08%, outperforming DenseNet-201 and SE-SqueezeNet, which achieved 96.48% and 95.70%, respectively. The proposed ensemble technique outperformed individual models, yielding an accuracy of 97.45%. In the next experiment, we used lung-segmented images instead of the whole CXR image to train the models. Following lung segmentation, all the models exhibited improvement as more relevant features were incorporated for classification. Additionally, the final ensemble model outperformed all the individual base models, demonstrating an accuracy of 98.15% and an F1-score of 97.89%. This outcome affirms the validity of Hypothesis 1. Sensitivity is a pivotal metric in disease classification, and the final ensemble model demonstrates a significant sensitivity level of 98.10%. Next, we performed four class classifications by adding the TB datasets with the existing three classes. Table 4 represents the outcome of 4-class classification models with and without segmentation. The outcomes indicate that initial segmentation has achieved 1.04% higher accuracy than without segmentation, which validates Hypothesis 2. We have utilized the Adam optimizer paired with categorical cross-entropy loss function and learning rate = 0.00001. The classification models were trained in mini-batches of 32 for a maximum of 100 epochs, employing an early stopping technique. Table 2 presents the outcome of infection segmentation models. UNet with VGG-16 encoder performs remarkably well, achieving a Dice score of 81.36%. Additionally, UNet with DenseNet-201 and ResNet-50 encoder produces competitive Dice scores of 80.44% and 81.07%, respectively. We combined the predicted masks from the top three individual networks and attained an outstanding Dice score of 96.75% and an IoU score of 93.71%. This significant enhancement validates the effectiveness of the ensemble approach. We have utilized the Adam optimizer with an initial learning rate of 0.0001 and trained the models in mini-batches of 8, continuing for a maximum of 100 epochs. All the above experiments are carried out on a local computer operating on Linux with 32GB of RAM and NVIDIA Quadro RTX 4000 GPU with 40GB of memory. Lung segmentation models required about 4 hours of training for each model, while classification models, including DenseNet 201 and SE-Inception-V3, took approximately 3 hours. SE-SqueezeNet models, on the other hand, required around 4 hours for training. Infection segmentation models were trained in approximately 2 hours.

**Table 2:** Lung Segmentation and Infection Segmentation results.

Model	Lung segmentation		Infection segmentation	
	IOU	Dice	IOU	Dice
UNet	**95.74**	**97.68**	72.48	75.37
UNet + VGG-16	**95.73**	**97.67**	**68.57**	**81.36**
Attention UNet	95.63	97.62	71.36	73.21
UNet + DenseNet-201	95.46	97.51	**67.28**	**80.44**
UNet + ResNet-50	95.72	97.67	**68.16**	**81.07**
UNet + Inception-V3	95.73	97.66	71.71	74.72
UNet + DenseNet-121	**95.86**	**97.74**	52.18	68.58
**Ensemble model**	**98.99**	**99.49**	**93.71**	**96.75**

Significant values are in bold.

**Table 3:** Experiment 1: 3-class classification.

	Model	Precision (%)	Sensitivity (%)	F1-score (%)	Accuracy (%)
	SE-SqueezeNet	94.99	95.52	95.25	95.70
Without segmentation	DenseNet 201	95.75	96.27	96.00	96.48
	SE-Inception-V3	96.68	97.04	96.85	97.08
	**Proposed Ensembled**	**96.94**	**97.36**	**97.13**	**97.45**
	SE-SqueezeNet	96.32	96.56	96.43	96.85
With segmentation	DenseNet 201	96.80	97.55	97.17	97.50
	SE-Inception-V3	97.34	97.80	97.56	97.87
	**Proposed Ensembled**	**97.69**	**98.10**	**97.89**	**98.15**

Significant values are in bold.

**Table 4:** Experiment 2: 4-class classification.

	Model	Precision (%)	Sensitivity (%)	F1-score (%)	Accuracy (%)
	SE-SqueezeNet	89.61	93.87	91.69	94.18
Without segmentation	DenseNet 201	92.83	95.32	94.05	96.05
	SE-Inception-V3	95.41	94.17	94.78	96.35
	**Proposed Ensembled**	**94.72**	**96.67**	**95.36**	**97.09**
	SE-SqueezeNet	94.23	96.34	95.27	96.78
With segmentation	DenseNet 201	97.06	97.58	97.31	98.04
	SE-Inception-V3	96.02	95.89	95.95	96.91
	**Proposed Ensembled**	**96.80**	**98.09**	**97.44**	**98.13**

Significant values are in bold.

**Figure 3:**
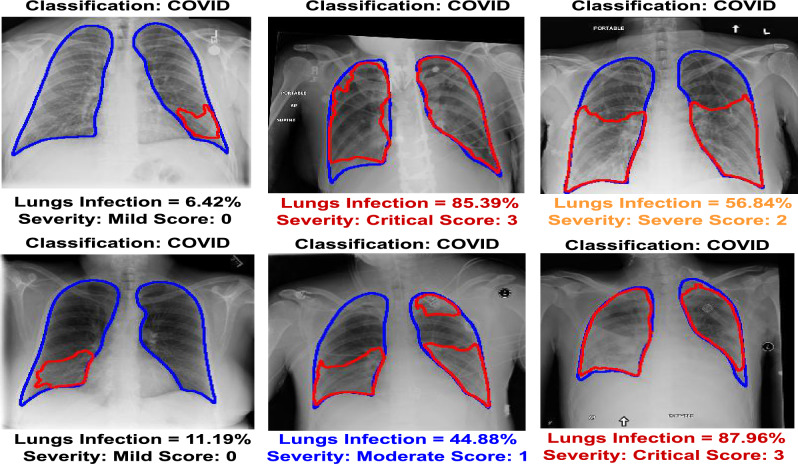
The outcome of the infection segmentation and severity assessment for COVID-19 samples. Blue areas are actual lung regions, and red areas are infection regions predicted by the model..

## Infection visualization and severity assessment

As mentioned, our proposed architecture utilizes the modified RALE score to assign a severity level according to the extent of infection in lung regions. These levels include mild, moderate, severe, and critical, as illustrated in Fig. 3. This approach aids medical professionals in prioritizing COVID-19-infected patients and devising effective treatment plans, particularly in resource-constrained environments. In Fig. 3, the first image represents a COVID-19 CXR image with a 6.42% lung infection, leading to classification as ”mildly infected.” Such patients may not require immediate admission. Conversely, the second image depicts a patient with an 85.39% infection, categorized as a ’critical patient’ necessitating urgent admission for treatment. This method significantly enhances clinical decision-making by accurately classifying COVID-19 patients from other respiratory diseases while also providing insight into the disease’s severity level. For a more comprehensive overview, please refer to the Supplementary File regarding Hypothesis 3.

## Comparative analysis

We rigorously assessed the proposed model by comparing its performance against recent state-of-the-art methods mentioned in the literature survey. To ensure an equitable comparison, we exclusively considered models for which detailed implementations are available in the publications. We have trained these models following the specifications mentioned in their respective papers with our datasets. Table 5 presents the proposed model’s results with six baseline models. Baseline-1 fine-tuned pre-trained CNNs and applied the Sugeno fuzzy ensemble to combine the outcomes. However, this method manually calculates fuzzy measure values, which is time-consuming. Baseline-2 enhanced the previous work by introducing a novel way of calculating fuzzy measure values from validation accuracy. Baseline-3 employed the Choquet fuzzy ensemble to combine the CNN outcomes. Baseline-4 adopted the weighted average ensemble technique to combine decisions from pre-trained classifiers, which require careful weight selection and assume independence among models, making them sensitive, complex, and potentially limited in handling diverse or non-numeric predictions. Baseline-5 utilized a vision transformer as a base model with federated training, and the authors in Baseline-6 utilized CNN models with one added LSTM layer with the Sugeno fuzzy ensemble. Both methods attain an accuracy of 97.14% and 97.42%, respectively, on our dataset. Baseline-7 utilized a rank-based ensemble utilizing two non-linear functions and reported an 98.05% accuracy on a three class classification problem. Contrastingly, our proposed model achieved a substantially higher accuracy of 98.15% for three-class classification and 98.13% for four-class classification, surpassing existing baseline models. The distinct advantage of our proposed gamma-based ensemble lies in its reduced reliance on individual model choices and employs a sophisticated decision-making process.

**Table 5:** Comparative analysis of our method with the existing state-of-the-art methods in the literature.

Method	Precision (%)	Sensitivity (%)	F1-score (%)	Accuracy (%)
Baseline-1^28^	95.22	95.21	95.21	95.43
Baseline-2^25^	96.87	97.22	97.04	97.31
Baseline-3 ^29^	97.03	97.49	97.25	97.54
Baseline-4 ^20^	96.38	97.01	96.69	96.99
Baseline-5^7^	96.97	97.08	97.02	97.14
Baseline-6^47^	97.18	97.24	97.21	97.42
Baseline-7^12^	97.58	97.96	97.76	98.05
**Proposed Method** (3-class classification)	**97.69**	**98.10**	**97.89**	**98.15**
**Proposed Method** (4-class classification)	**96.80**	**98.09**	**97.44**	**98.13**

Significant values are in bold.

## Additional experiment on unseen dataset

The assessment of an end-to-end model must be generalizable to different datasets with different patient groups. Developing a reliable and clinically effective model is crucial for any robust model development. In this study, we evaluated the proposed architecture using a different test set comprising diverse patient groups, sourced from the widely-used QaTa-COV19 dataset^39^, which includes 2913 CXR samples of COVID-19 cases. The proposed architecture achieves 97.89% accuracy with an F1-score of 97.48%. The model’s outcomes remain consistent despite variations in the dataset because it concentrates explicitly on pertinent lung regions. This indicates the model’s ability to perform effectively on new and diverse datasets, even with limited training data.

## Discussion

Similar findings of common respiratory diseases in CXR images make it challenging and error-prone for medical professionals. This research presents a comprehensive, multi-stage architecture to support better clinical decision-making for patient outcomes and alleviate hospital staff workloads. The proposed architecture consists of a three-stage process involving a three-stage process encompassing lung segmentation, disease classification, infection localization, and severity assessment. In order to reduce information loss during lung segmentation, we tested different UNet variants and combined the output masks from the three models that performed the best. Three pre-trained CNN models are utilized for classification, followed by the proposed ensemble model that generates the fuzzy ranks of the classifiers using the gamma function. By using the gamma function, the proposed method is able to more effectively combine the predictions of different models, leading to improved accuracy and performance compared to other ensemble methods. Moreover, when validated with an extra dataset, the suggested technique proves its reliability, remaining resilient against the diverse nature of different datasets. This robustness stems from the model’s focus on pertinent lung regions during decision-making. During the last phase, the model detects infected regions, assigns a score, and determines the corresponding infection level whenever the input CXR image is COVID-19. We calculate the score function by modifying the RALE scoring method. The optimistic outcomes of the suggested approach can aid radiologists in understanding the disease progression and devising more effective treatment strategies in the early stages. In conclusion, while the outcomes of our study are promising, there are a few challenges. Firstly, the complexity of the ensemble model is higher than that of the standalone architectures, leading to a higher computational complexity. Further research on adopting lightweight pre-trained models in the ensemble process could mitigate the computational complexity. Second, the reliance on additional training data for pre-trained models poses a limitation, prompting further investigation into the model’s generalizability in few-shot dataset settings. Lastly, while we focused on three prevalent lung diseases in our proposed work, extending the research to encompass other common respiratory diseases is essential for practical diagnostic applicability.

### Supplementary Information

Supplementary Information.

## Data Availability

The source code can be accessed at https://github.com/Pranabiitp/Boosting-Interpretability-and-Generalization-of-Respiratory-Disease-from-CXR-image. The curated COVID-19 dataset is available at our github https://github.com/Pranabiitp/COVID-19-CXR-Dataset. All the other datasets are publicly available.
