# Supplementary material for: A multistage framework for respiratory disease detection and assessing severity in chest X-ray images
Source: Sci Rep. 2024 May 29;14:12380. doi: 10.1038/s41598-024-60861-6 (PMC11137152; doi:10.1038/s41598-024-60861-6)
Supplement: Supplementary file 1 — Supplementary Information. [file 41598_2024_60861_MOESM1_ESM.pdf]

A Multistage Framework for Respiratory Disease Detection and Assessing Severity in Chest X-ray Images

Pranab Sahoo[1,*], Saksham Kumar Sharma[2], Sriparna Saha[1], Deepak Jain[3], and Samrat Mondal[1]

[1]Department of Computer Science & Engineering, Indian Institute of Technology Patna, 801106, India
[2]Maharaja Surajmal Institute of Technology, Delhi, India
[3]Mount Sinai Hospital and Icahn School of Medicine, New York, USA
[*]pranab_2021cs25@iitp.ac.in

ABSTRACT

This supplementary file contains all the related information.

1 Related Works

Ref.	Imaging modalities	Lung Segmentation	Architecture	Infection Segmentation	Severity Assessment
Wang et al.[1]	CXR	✗	Weakly supervised CNN	✓	✗
Rajpurkar et al.[2]	CXR	✗	CheXNet	✓	✗
Rahman et al.[3]	CXR	✓	Transfer Learning	✓	✗
Nishio et al.[?]	CXR	✗	Transfer Learning	✗	✗
Wang et al.[4]	CXR	✗	COVID-Net	✗	✗
Kaleem et al.[?]	CXR	✗	Ensemble Learning	✗	✗
Chandra et al.[5]	CXR	✗	Majority vote based Ensemble	✗	✗
Gifani et al.[6]	CT	✗	Majority vote based Ensemble	✗	✗
Das et al.[7]	CXR	✗	Weighted average Ensemble	✗	✗
Kundu et al.[8]	CT-Scan	✗	Sugeno Fuzzy based Ensemble	✗	✗
Dey et al.[9]	CXR	✗	Choquet Fuzzy based Ensemble	✗	✗
Sahoo et al.[10]	CT-Scan	✗	Modified Sugeno Fuzzy Ensemble	✗	✗
Xu et al.[11]	CXR	✓	Mask attention based CNN	✗	✗
Sharma et al.[12]	CXR	✓	Transfer Learning with attention	✓	✓
Warren et al.[13]	CXR	✗	RALE Scoring System	✓	✓
Borghesi et al.[14]	CXR	✗	Brixia Score System	✓	✓
. Toussie et al.[15]	CXR	✗	Array-based Scoring	✗	✓

Table 1. A comparative summary of the related works

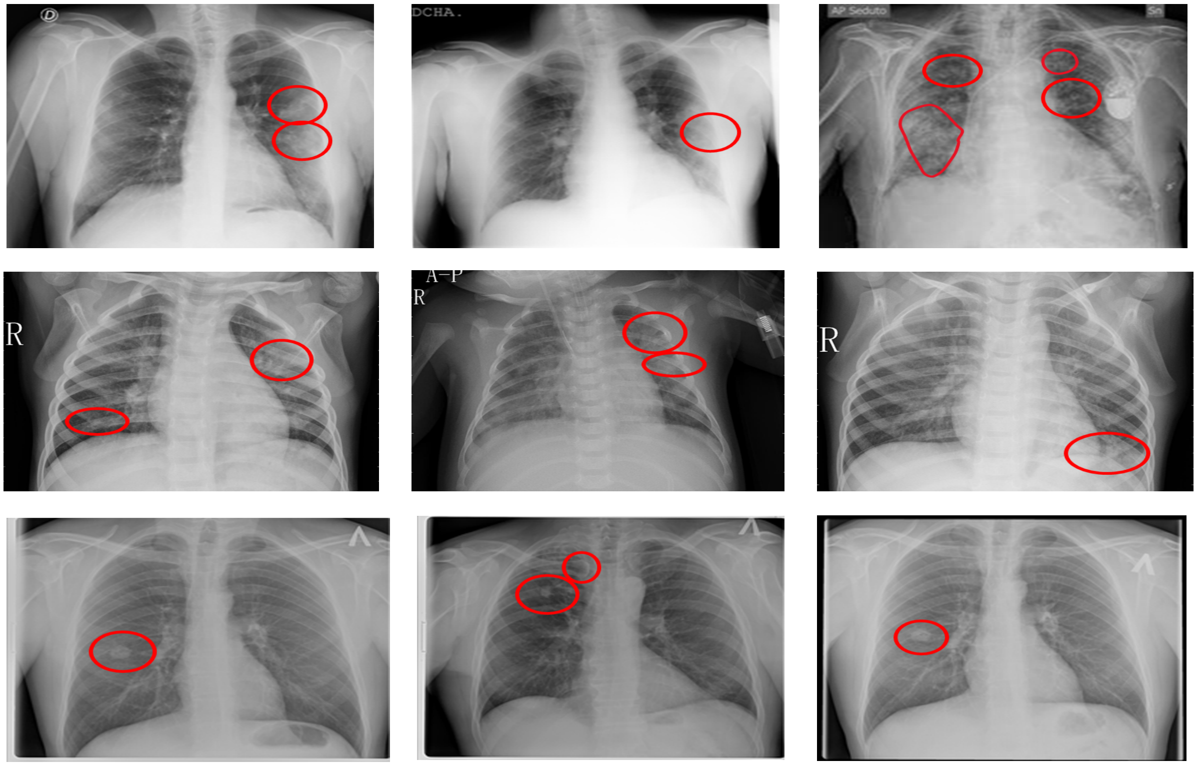

Figure 1. Example of the sample images. The first row is the COVID-19 images, the second row is the pneumonia images, and the third row is the TB images. Findings are marked by the radiologist.

2 Dataset

Some of the Radiologist marking images from the dataset are shown in Fig. 1.

3 Algorithm

Input : Number of base models: m,
Number of classes: c,
Confidence scores: $X_1^m, X_2^m, X_3^m, \ldots, X_c^m$
Output : Final predictions: class(I)

for $(i = 1; i <= m; i++)$ **do**
  for $(k = 1; k <= c; k++)$ **do**
    $R_k^i = \Gamma(X_k^i)$
    $FRS_c = \sum_{i=1}^{M} R_k^i$
    $CCFS_k = \frac{1}{M} \sum (1 - X_k)^i$
    $FDS_k = FRS_k \times CCFS_k$
  end
end
$class(I) = argmax_k(FDS_k)$

Algorithm 1: Pseudo code for calculating Fuzzy rank

4 Hypothesis-3

Hypothesis 3: The infection segmentation module could improve the interpretability of the model rather than the Grad-CAM-based approach. To prove this, we analyzed Grad-CAM results with radiologist markings along with infection segmentation model outputs.

Grad-CAM techniques have been widely used for visualizing and interpreting deep neural networks. However, we have observed some limitations while working with the chest x-ray images. (1) The generated heatmaps do not precisely identify the exact boundaries of infections, making it challenging to pinpoint specific details. (2) Grad-CAM faces difficulties distinguishing

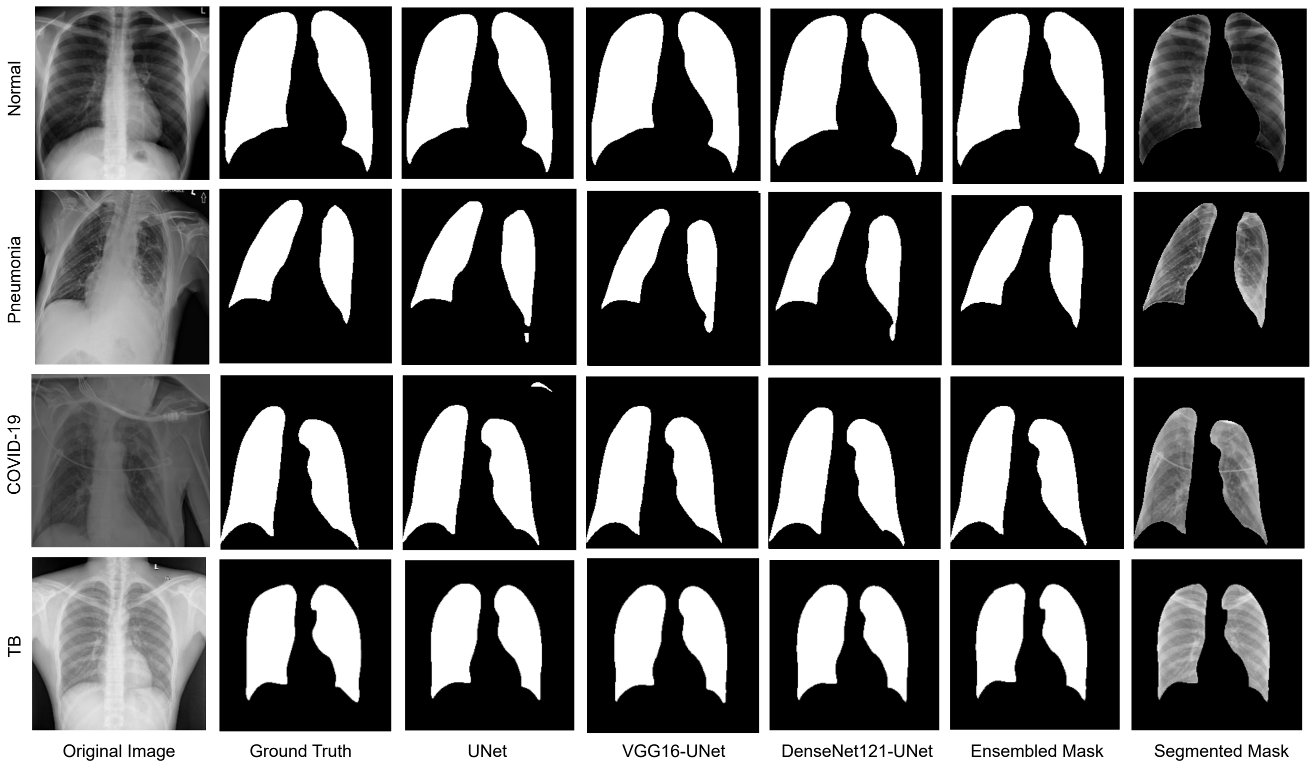

Figure 2. The predictions of lung segmentation network

between closely related COVID-19 and Pneumonia classes. It generates heatmaps highlighting features shared between classes, making it challenging to interpret the infected regions. (3) Based on the experiments, we have observed that it also highlights some parts that are not the lung regions.

We have validated the the proposed modified RALE scoring system in consultation with one medical expert who has more than 5 years of experience in the radiology field and is working at Mount Sinai Hospital and Icahn School of Medicine, New York, USA. The scoring system was tested randomly on 50 COVID-19 CXR images. The automated scoring system's predictions are then compared with these ground truth labels generated by the radiologist.

Fig. 3 shows one instance where the Grad-CAM generated regions are not comparable with radiologist markings. However, the infection segmentation models produce accurate localization. This validates our hypothesis 3.

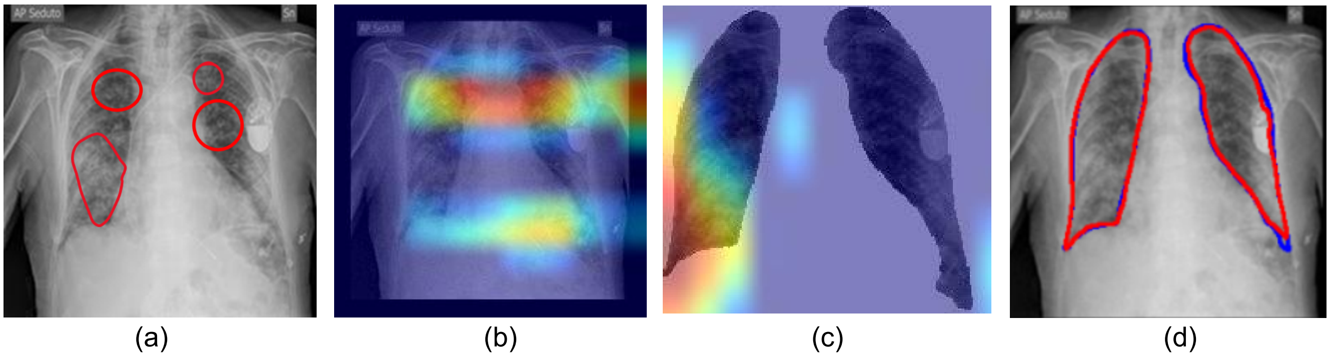

Figure 3. The predictions of lung segmentation network

References

1. Wang, X. *et al.* Chestx-ray8: Hospital-scale chest x-ray database and benchmarks on weakly-supervised classification and localization of common thorax diseases. In *Proceedings of the IEEE conference on computer vision and pattern recognition*, 2097–2106 (2017).
2. Rajpurkar, P. *et al.* Chexnet: Radiologist-level pneumonia detection on chest x-rays with deep learning. *arXiv preprint arXiv:1711.05225* (2017).
3. Rahman, T. *et al.* Reliable tuberculosis detection using chest x-ray with deep learning, segmentation and visualization. *IEEE Access* **8**, 191586–191601 (2020).
4. Wang, L., Lin, Z. Q. & Wong, A. Covid-net: A tailored deep convolutional neural network design for detection of covid-19 cases from chest x-ray images. *Sci. Reports* **10**, 1–12 (2020).
5. Chandra, T. B., Verma, K., Singh, B. K., Jain, D. & Netam, S. S. Coronavirus disease (covid-19) detection in chest x-ray images using majority voting based classifier ensemble. *Expert. systems with applications* **165**, 113909 (2021).
6. Gifani, P., Shalbaf, A. & Vafaeezadeh, M. Automated detection of covid-19 using ensemble of transfer learning with deep convolutional neural network based on ct scans. *Int. journal computer assisted radiology surgery* **16**, 115–123 (2021).
7. Das, A. K. *et al.* Automatic covid-19 detection from x-ray images using ensemble learning with convolutional neural network. *Pattern Analysis Appl.* 1–14 (2021).
8. Kundu, R., Singh, P. K., Mirjalili, S. & Sarkar, R. Covid-19 detection from lung ct-scans using a fuzzy integral-based cnn ensemble. *Comput. Biol. Medicine* **138**, 104895 (2021).
9. Dey, S., Bhattacharya, R., Malakar, S., Mirjalili, S. & Sarkar, R. Choquet fuzzy integral-based classifier ensemble technique for covid-19 detection. *Comput. Biol. Medicine* 104585 (2021).
10. Sahoo, P., Saha, S., Mondal, S., Chowdhury, S. & Gowda, S. Computer-aided covid-19 screening from chest ct-scan using a fuzzy ensemble-based technique. In *2022 International Joint Conference on Neural Networks (IJCNN)*, 1–8 (IEEE, 2022).
11. Xu, Y., Lam, H.-K. & Jia, G. Manet: A two-stage deep learning method for classification of covid-19 from chest x-ray images. *Neurocomputing* **443**, 96–105 (2021).
12. Sharma, A. & Mishra, P. K. Covid-manet: Multi-task attention network for explainable diagnosis and severity assessment of covid-19 from cxr images. *Pattern Recognit.* 108826 (2022).
13. Warren, M. A. *et al.* Severity scoring of lung oedema on the chest radiograph is associated with clinical outcomes in ards. *Thorax* **73**, 840–846 (2018).
14. Borghesi, A. & Maroldi, R. Covid-19 outbreak in italy: experimental chest x-ray scoring system for quantifying and monitoring disease progression. *La radiologia medica* **125**, 509–513 (2020).
15. Toussie, D. *et al.* Clinical and chest radiography features determine patient outcomes in young and middle-aged adults with covid-19. *Radiology* **297**, E197 (2020).
